# Novel Nematode-Killing Protein-1 (Nkp-1) from a Marine Epiphytic Bacterium *Pseudoalteromonas tunicata*

**DOI:** 10.3390/biomedicines9111586

**Published:** 2021-10-30

**Authors:** Nor Hawani Salikin, Malak Dubois, Jadranka Nappi, Helene Lebhar, Christopher Marquis, Suhelen Egan

**Affiliations:** 1 Centre for Marine Science and Innovation, School of Biological, Earth and Environmental Sciences UNSW, Sydney, NSW 2052, Australia; j.nappi@unsw.edu.au; 2 School of Industrial Technology, Universiti Sains Malaysia, USM, Gelugor 11800, Penang, Malaysia; 3 School of Biotechnology and Biomolecular Sciences, UNSW, Sydney, NSW 2052, Australia; malak.dubois@det.nsw.edu.au (M.D.); h.lebhar@unsw.edu.au (H.L.); c.marquis@unsw.edu.au (C.M.)

**Keywords:** anti-nematode, anthelminthic drugs, bioactives, *Pseudoalteromonas tunicata*, nematode, *Caenorhabditis elegans*, toxic protein

## Abstract

Drug resistance among parasitic nematodes has resulted in an urgent need for the development of new therapies. However, the high re-discovery rate of anti-nematode compounds from terrestrial environments necessitates a new repository for future drug research. Marine epiphytes are hypothesised to produce nematicidal compounds as a defence against bacterivorous predators, thus representing a promising yet underexplored source for anti-nematode drug discovery. The marine epiphytic bacterium *Pseudoalteromonas tunicata* is known to produce several bioactive compounds. Screening heterologously expressed genomic libraries of *P. tunicata* against the nematode *Caenorhabditis elegans*, identified as an *E. coli* clone (HG8), shows fast-killing activity. Here we show that clone HG8 produces a novel nematode-killing protein-1 (Nkp-1) harbouring a predicted carbohydrate-binding domain with weak homology to known bacterial pore-forming toxins. We found bacteria expressing Nkp-1 were able to colonise the *C. elegans* intestine, with exposure to both live bacteria and protein extracts resulting in physical damage and necrosis, leading to nematode death within 24 h of exposure. Furthermore, this study revealed *C. elegans* dar (deformed anal region) and internal hatching may act as a nematode defence strategy against Nkp-1 toxicity. The characterisation of this novel protein and putative mode of action not only contributes to the development of novel anti-nematode applications in the future but reaffirms the potential of marine epiphytic bacteria as a new source of novel biomolecules.

## 1. Introduction

Diseases resulting from parasitic helminth (nematode) infections are a global concern, especially for resource-limited countries with poor hygiene and an inadequate clean water supply [1,2]. Globally, approximately 1.5 billion people suffer from parasitic helminth infections, with the majority of cases occurring in East Asia, sub-Saharan Africa, America and China [3]. In humans, parasitic nematode infections are associated with malnutrition, anaemia, growth retardation, diminishing fitness, reduced cognition and result in numerous fatalities per year [2,4]. In addition to human illness, parasitic nematodes can also infect agricultural crops and aquaculture products, hence reducing yield and threatening food security [5,6]. 

For the past 40 years, strategies to control parasitic nematodes have almost exclusively relied on intensive chemotherapy to relieve symptoms and diminish transmission. However, overuse and frequent parasite exposure to single broad-spectrum therapeutic drugs can increase the prevalence of anthelmintic resistance among harmful parasites [7,8,9] and therefore finding new anthelmintic compounds has become a matter of global urgency. The nematode *Caenorhabditis elegans* has been widely used as the surrogate organism for drug screening, as it shares many conserved genes and protein function with parasitic nematodes [10,11] yet is easily maintained in the laboratory [12,13]. 

Whilst the search for new candidate anthelmintic drugs is ongoing, marine-derived bioactive compounds particularly from surface-associated microbiota are a promising resource [14,15]. Bacteria from the genus *Pseudoalteromonas* have been recognised for their ability to produce a range of commercially and pharmaceutically relevant bioactivities against micro- and macrofoulers [16,17]. *P. tunicata* D2 is arguably the most comprehensively studied microorganism within the genus owing to its production of low- and high-molecular-weight compounds that are toxic against a wide spectrum of environmentally and medicinally relevant organisms including nematodes [18,19]. Functional screening of a *P. tunicata* D2 genomic fosmid library discovered a heterologous clone (designated HG8) that was toxic against the nematode *C. elegans* [18]. Genetic characterisation of HG8 revealed that a gene of unknown function (NCBI GeneBank Accession Number ZP_01132244.1) was partly responsible for its anti-nematode activity [18]. However, the heterologous expression of ZP_01132244.1 alone did not show significant anti-nematode activity (Ballestriero et al., unpublished data), suggesting that other genetic determinants are required for the nematode-killing activity. 

Given the need for new anti-nematode drugs, the current study aimed to characterise and identify the bioactive agent responsible for the anti-nematode activity of the clone HG8 and its MOAs against *C. elegans*. Screening of the HG8 transposon mutant library identified a mutant of the HG8 clone (designated 7C8) showing attenuated anti-nematode activity due to the disruption of an uncharacterised gene designated *HP1* (NCBI GeneBank Accession Number ZP_01132246.1). Analyses of the active clones revealed *HP1* encodes for a novel protein designated nematode-killing protein-1 (Nkp-1) that harbours an N-terminal carbohydrate-binding module and has a putative mode of action similar to cry-like or aerolysin-like bacterial toxins. The discovery of this novel anti-nematode protein will hopefully contribute to the development of new therapies from the ocean that can be applied to parasitic nematode control management in the future.

## 2. Materials and Methods

### 2.1. Bacterial Strains and Culture Conditions

All bacterial strains and vectors used in this study are listed in Table 1. Unless otherwise stated, *E. coli* strains and other bacterial isolates were grown in lysogeny broth (LB10) and nematode growth media (NGM) [20] and were kept in 30% (*v*/*v*) glycerol at −80 °C. Solid media was prepared with the addition of 1.5% (*w*/*v*) of agar (Oxoid, Australia). Where required, L-(+)-arabinose (0.2% *w*/*v*) and antibiotics such as chloramphenicol (12.5 µg/mL), ampicillin (50 µg/mL) and kanamycin (50 µg/mL) were incorporated into the media.

### 2.2. Maintenance and Synchronisation of Nematode C. elegans

N2 strain Bristol *C. elegans* were maintained on NGM seeded with *E. coli* OP50 as the food source [20] and synchronised using fresh bleaching solution according to Stiernagle et al. [25].

### 2.3. Generation of HG8 Mutant Libraries and Screening against C. elegans

Transposon mutagenesis of the HG8 fosmid clone was performed using the EZ-Tn5™<T7/KAN-2> promoter Insert Kit (Epicentre, Madison, WI, USA) as per the manufacturers’ instructions. Successful mutants were screened on LB10 agar with chloramphenicol and kanamycin. Selected mutants were grown overnight in 96-well plates (Becton Dickinson Co Biosciences, Bedford, MA, USA) containing LB10 with antibiotics at 37 °C, 60 rpm. Mutant libraries were replicated onto omnitory plates (Nunc brand, Germany) containing LB10 agar with antibiotics and L-(+)-arabinose (Sigma-Aldrich, St. Louis, MO, USA) using a 96 Solid Pin Multi-Blot Replicator tool (V&P Scientific Inc.) followed by 48 h of incubation at 25 °C. Synchronised L4 stage *C. elegans* (30–40 nematodes) were transferred onto the plate for selective grazing against the mutant libraries in triplicate. Colonies that were completely grazed after 4 days were then subject to a nematode-killing assay (described below) with the wild-type HG8 clone and a randomly selected non-toxic strain from the original *P. tunicata* fosmid library (BG6) [18] as the positive and negative controls, respectively. Non-toxic HG8 mutants resulting in >60% *C. elegans* survival in the nematode-killing assay were sequenced bi-directionally by the Ramaciotti Centre for Genomics (University of New South Wales, Sydney, AU) for transposon mapping using the EZ-Tn5™<T7/KAN-2> promoter Insert Kit (Epicentre) Forward (5′ ACCTACAACAAAGCTCTCATCAACC 3′) and Reverse (5′GCAATGTAACATCAGAGATTTTGAG 3′) primers. 

### 2.4. Cloning of P. tunicata Gene (HP1) into pBAD24 Vector

The *HP1* gene was amplified by PCR containing forward; 5′ ATGAGTACTACAATTTGGAACGATG 3′ and reverse primers; 5′ CTGGCTTGTTATCGCCATTT 3′ (10 pmol), DNA template (HG8 fosmid; 20 ng), Phusion^®^ High-Fidelity PCR Master Mix with HF Buffer (NEB) and nuclease-free water (Ambion^®^ Austin, TX, USA) up to 50 µL of reaction volume and were run under a thermocycler condition of 98 °C for 30 s, 30 cycles of initial denaturation at 98 °C for 30 s, annealing at 60 °C for 30 s, extension at 72 °C for 30 s and a final extension at 72 °C for 7 min. The amplicons were purified and phosphorylated using the T4 Polynucleotide Kinase (NEB) prior to blunt-ended ligation to the linearized pBAD24 vector, blunt-end repaired by the DNA Polymerase I, Large (Klenow) Fragment (NEB) and dephosphorylated at the 5′ end using the Shrimp Alkaline Phosphatase (rSAP) (NEB) as per the manufacturer’s instructions. The phosphorylated amplicons were ligated alongside the pBAD24-NcoI-Klenow-rSAP treated vector using the T4 DNA ligase (NEB) and were transformed into the initially prepared electrocompetent *E. coli* EPI300 cells [26]. Transformants were recovered in SOC media and screened for successful recombinant clones on LB10 agar with ampicillin. Positive clones with correct insert DNA orientation were established by colony-pick PCR and submitted for sequencing analysis by the Ramaciotti Centre for Genomics, UNSW, Australia. Next, the recombinant vectors were transformed into the initially prepared electrocompetent 7C8 mutant cells [26] and were screened on LB10 agar with chloramphenicol, kanamycin and ampicillin. Colony-pick PCR was used to validate successful complementation and transformants were challenged against *C. elegans* using the nematode-killing assay.

### 2.5. Nematode-Killing Assay

Assay plates were seeded with an overnight culture of test bacteria (30 µL) onto 35 mm × 10 mm plates (SARSTEDT) containing LB10 agar with appropriate antibiotics and L-(+)-arabinose and were incubated at 25°C for four days. Thirty to forty synchronised L4 stage *C. elegans* individuals were transferred onto the test bacterial lawn and incubated at 25 °C. To assess *C. elegans* survival against the HP1 and HG8 crude protein extracts, a liquid anti-nematode assay was carried out in 24-well microtiter plates [27]. Wells were incorporated with 150 µL of each protein extract (equivalent to ~0.7 mg of total protein content) supplemented with 40 µL of concentrated *E. coli* OP50 (OD600 nm = 2.0) and M9 buffer up to a final volume of 300 µL. Twenty to thirty L4 stage nematodes were transferred into each well of the 24-well microtiter plate and incubated in 25 °C for 72 h. The proportion of live nematodes were recorded every 24 h under the dissecting stereomicroscope (Olympus SZ-CTV). Nematodes were considered dead when they showed no response to touch (using sterilised worm picker). A test bacterial strain was considered toxic to *C. elegans* when more than 50% of the assayed nematodes were killed within 3 days [28]. Experiments were performed in triplicate and monitored until the maximum lifespan of the non-toxic control either *E. coli* OP50, *E. coli* EP300 or *E. coli* BD24 was reached.

### 2.6. Egg Hatching and Brood Size Assay

Assessment of *C. elegans* egg hatching and brood size following HP1, HG8 and BD24 bacterial exposure was investigated as described previously [29,30]. Nematode eggs were harvested via synchronisation [25] and eggs were pelleted through centrifugation at 1000× *g* for 10 min and resuspended in 1 mL of M9 buffer. Next, ~60 eggs were inoculated onto each assay plate and incubated at 25 °C for 7 h. Numbers of L1 hatchlings on each plate were quantified under the dissecting microscope. To investigate the effect of HP1 and HG8 on *C. elegans* brood size, a single L4 stage nematode was transferred onto a nematode-killing assay plate containing a lawn of the test bacteria. The number of nematode progenies arising from an individual was quantified after 72 h incubation at 25 °C. The experiment was performed in triplicate. 

### 2.7. Bacterial Colonisation Assay

HP1 and HG8 bacterial colonisation of *C. elegans* was determined as previously described [31,32]. L4 stage nematodes were washed from the NGM plates using the M9 buffer and were exposed to HP1::GFP, HG8::GFP and BD24::GFP strains on the LB10 agar. Thirty nematodes were picked from plates for each treatment (i.e., 10 worms/plate in triplicate) and used for microscopy analysis. The intensity of fluorescence signals from the acquired images were quantified using ImageJ (http://rsbweb.nih.gov/ij/download.html. Accessed on 5 April 2020). Any observed morphological changes in *C. elegans* body structure, e.g., size reduction, pharynx distortion, degenerated internal organ, internal hatching or deformed anal region (dar), were recorded. To count the number of bacterial cells that colonised *C. elegans*, 10 live nematodes were picked after 6, 12, 24, 48, 72 and 96 h of assay and transferred onto the LB10 agar with gentamicin (100 µg/mL) to kill the surface-associating bacteria. Nematodes were washed with 10 µL of LM buffer (60 µg/mL levamisole in M9 buffer) and then washed twice in LM buffer containing 100 µg/mL gentamicin and M9 buffer alone. The removal of surface bacteria was confirmed by inoculating the final M9 buffer wash onto the LB10 agar plates following an overnight incubation at 37 °C. Non-viable bacteria on the LB10 media indicated the successful removal of bacteria from the nematode surface. Washed *C. elegans* were transferred into a 1.5 mL microcentrifuge tube containing 200 µL of phosphate buffer saline (PBS) with 1% Triton X-100 and mechanically disrupted using a sterilized pestle. Nematode lysates were serially diluted in PBS and spread plated onto the LB10 agar with appropriate antibiotics. After an overnight incubation at 37 °C, the number of colonies were quantified and represented as colony-forming unit per nematode (CFU/nematode). Each experiment was performed in triplicate.

### 2.8. Enzymatic Assay

HP1, HG8 and BD24 overnight cultures and their protein extracts were assayed for a range of hydrolytic enzyme activities. Where relevant the bacterium *Pseudomonas aeruginosa*, which actively produces protease [33], lipase [34], gelatinase (collagenase) [35] and chitinase [36], was included as the positive control. LB10 broth or PBS was used as the negative control. Protease, lipase, gelatinase/collagenase and chitinase activities were assayed on prepared skim milk agar [37], gelatine agar [38], Tween 80 agar [39] and chitin agar [40], respectively. Fifty microlitres of bacterial cultures or their protein extracts were inoculated into the agar wells made using a sterilised cork borer (diameter size 8 mm). Plates were incubated for 48 to 96 h at 25 °C. Positive enzymatic activity was indicated by the clearance zone observed surrounding the well. Each experiment was performed in triplicate.

### 2.9. Necrosis Assay

*C. elegans* exposed to HP1, HG8 and BD24 bacteria were assayed for necrotic cell death as described elsewhere [41,42]. Synchronized L4 stage *C. elegans* were washed from the *E. coli* OP50 lawn using the M9 buffer. Seventy to eighty nematodes were transferred onto the test bacterial lawn followed by incubation at 25 °C for 48 h. At least 30 nematodes per treatment (10 nematodes/replicate plate) were picked from the triplicate test bacterial lawns and washed twice in 20 µL of M9 buffer. Nematodes were transferred into each well of a 96-well microtiter plate containing 10 µM propidium iodide (PI) (Sigma Aldrich) in an M9 buffer and incubated in the dark at 25 °C for three hours. The animals were subsequently washed with M9 buffer and observed using a fluorescence microscope. *C. elegans* was confirmed as having necrosis when the dye was visualised in cells adjacent to the intestinal lumen due to loss of membrane cell integrity. The number of *C. elegans* showing necrosis were quantified. Each experiment was performed in triplicate. 

### 2.10. Microscopy Imaging

*C. elegans* images were acquired using an Olympus BX61 fluorescence microscope equipped with cellSens Dimension microscopy imaging software (Olympus). Slides containing live nematodes were prepared as previously described [43]. *C. elegans* were mounted onto the agarose pad (2% *w*/*v* in M9 buffer) using a sterilised worm picker and anesthetised with 10 µL of levamisole (60 µg/mL in M9 buffer). To visualise the GFP-tagged bacterial colonisation within *C. elegans*, the Fluorescein Isothiocyanate (FITC) filter (excitation wavelength: 495 nm, emission wavelength: 519 nm) was used. Any morphological changes in the nematodes due to bacterial cells or their protein extracts exposure were inspected under the Differential Interference Contrast (DIC) filter. To determine any necrotic cell death, nematodes were observed under the Cyanine-3 (Cy3) filter (excitation wavelength: 550 nm, emission wavelength: 570 nm).

### 2.11. Protein Extraction, SDS PAGE and Protein Modelling 

The HP1, HG8 and BD24 bacteria were cultured in 2 x yeast tryptone (2YT) broth with appropriate antibiotics. The 2YT culture media was seeded with a 0.6% (*v*/*v*) bacterial overnight culture, grown shaking (200 rpm) at 37 °C until the OD (600 nm) = 0.6 and thereafter heterologous gene expression was induced with L-arabinose at 25 °C and 200 rpm shaking condition for 18 h. Cells were harvested via centrifugation at 6000× *g* for 7 min in 4 °C, washed with an equal volume of ice-cold phosphate buffer saline (PBS) and kept in −80 °C. Each of the cell pellets were resuspended in PBS and mechanically disrupted through ultra-sonication (Consonic, Australia) on ice for three minutes at 50% amplitude with 2 s on and off interval. The resulting cell lysate was spun at 10,000× *g* for 30 min at 4 °C. The supernatant-containing soluble cell fraction was removed and stored at 4 °C until further use. The remaining pellets (containing insoluble proteins) were washed twice each with ice cold Wash Buffer A (Triton 100 0.5% *v*/*v*, Tris 5 mM, EDTA 50 mM, pH 8.0) and Wash Buffer B (glycerol 0.5% *v*/*v*, Tris 5 mM, EDTA 50 mM, pH 8.0) before a double final wash with PBS at 4 °C. The insoluble protein fraction was resuspended in PBS and assayed for anti-nematode activity using the 24-well microtiter plate assay method. Total protein content in each fraction was analysed using the Lowry method and quantified based on the standard curve generated from a serially diluted Bovine Serum Albumin (BSA) [44]. SDS PAGE was performed according to Laemmli’s method [45,46] using 12% polyacrylamide precast gel (BioRad). Protein bands of interest were excised from the gel and sent to the Bioanalytical Mass Spectrometry Facility (BMSF), University of New South Wales, Australia, for trypsin digestion and LC-MS analysis using ESI-TRAP. The peptide mass values were used for protein identification based on a MASCOT database search [47]. Advanced protein modelling, the prediction of ligand binding sites and intensive protein analysis by computational method was performed using Phyre2 [48] and SWISS-MODEL [49].

### 2.12. Statistical Analysis

The ordination of data and statistical analysis were performed using GraphPad Prism software 8.3.0 (GraphPad Software, La Jolla, CA, USA). *C. elegans* survival was analysed using the log-rank (Mantel–Cox) method [50,51] and a one-way ANOVA followed by Tukey’s pairwise comparison test. Results for egg hatching, nematode brood size, bacterial colonisation and necrosis assay were analysed using a one-way ANOVA and Tukey’s pairwise comparison test whilst the proportion of nematodes with morphological changes due to bacterial exposure and their protein treatment was analysed using a two-way ANOVA followed by Tukey’s and Sidak’s test. All results are presented as the means ± standard error from triplicate samples. A *p*-value < 0.05 was considered significant. 

## 3. Results

### 3.1. Generation of HG8 Transposon Mutants and Restoration of 7C8 Mutant Attenuated Activity upon Complementation with HP1

**Figure 1:**
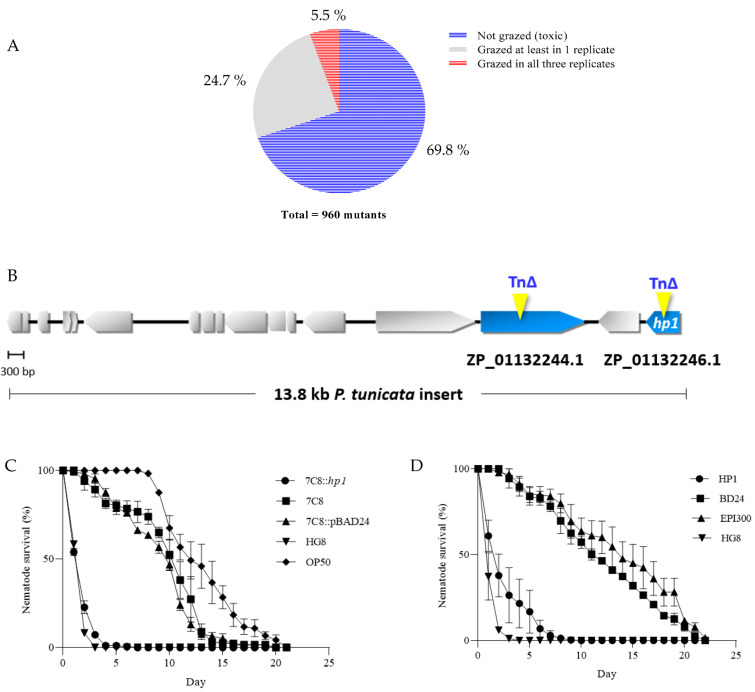
(**A**) The fraction of HG8 mutant libraries showing different levels of toxicity against *C. elegans*. (**B**) Transposon mutation in hypothetical proteins ZP_01132244.1 [18] and ZP_01132246.1 (denoted *HP1*) resulted in attenuated activity of HG8. (**C**) Complementation of 7C8 mutant with *HP1* successfully restored the anti-nematode activity whilst in (**D**), individual expression of *HP1* was also toxic against *C. elegans* compared to the non-toxic control BD24 and EPI300 bacterial strains. Significant differences among the treatments were detected (log-rank (Mantel–Cox) test; *p* < 0.0001). Each data point represents means of nematode survival ± standard error from triplicate samples.

A total of 960 HG8 transposon mutant clones were successfully generated and screened for diminished toxicity against *C. elegans*. In total, 69.8% (670 mutants) were not grazed by nematodes, 24.7% (237 mutants) were grazed at least in one replicate and the remaining 5.5% (53 mutants) were completely grazed in all three replicates (Figure 1A). To confirm the loss of anti-nematode activity for the grazed HG8 transposon mutants, 44 randomly chosen mutants from those that were grazed in at least one replicate were assessed for toxic activity using the nematode-killing assay. Approximately half (21/44) of these mutants were considered non-toxic with >60% nematode survival after 5 days. Five of the confirmed non-toxic mutants (denoted 8E10, 7C8, 1B11, 3B4 and 8D11) were randomly selected for transposon mapping. Sequencing of the DNA region directly adjacent to the transposon-insertion site revealed that mutant 8E10 was disrupted in the gene ZP_01132244.1 (Figure 1B), whilst mutants 7C8, 1B11, 3B4 and 8D11 were all disrupted in the neighbouring gene (NCBI Accession; ZP_01132246.1) which was then annotated as *HP1* (Figure 1B). *C. elegans* exposed to the mutant clone expressing a functional *HP1* in trans had reduced survival after 2 days compared to those exposed to the 7C8 mutant strain (one-way ANOVA followed by Tukey’s test; F (4, 10) = 239.9; *p* < 0.0001, Figure 1C) and a similar response as those exposed to the original toxic HG8 clone (one-way ANOVA followed by Tukey’s test; F (4, 10) = 239.9; *p* = 0.0356, Figure 1C). 

### 3.2. Expression of Individual HP1 in E. coli Reduced C. elegans Survival

To further validate the toxicity expressed by the *HP1* gene, the HP1 bacterial strain was assessed for its nematicidal activity against *C. elegans*. Exposure to HP1 after two days resulted in significant reduction in nematode survival compared to nematodes exposed to the non-toxic BD24 (*p* = 0.0006) and EPI300 control strains (*p* = 0.0007) (one-way ANOVA followed by Tukey’s test; F (3, 8) = 53.10, Figure 1D). These observations further validated the toxicity of *HP1* against *C. elegans*.

### 3.3. Exposure to HP1 and HG8 Does Not Affect C. elegans Eggs’ Hatching Efficiency but Decreases the Brood Size

**Figure 2:**
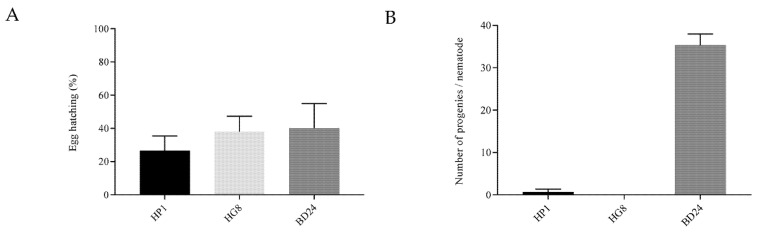
Effect of HP1, HG8 and BD24 bacterial exposure on (**A**) *C. elegans* egg hatching efficiency and (**B**) *C. elegans* brood size. No significant difference was observed on the proportion of *C. elegans* eggs hatching on HP1 and HG8 compared to BD24 bacterial strains (*p* > 0.05). However, the number of *C. elegans* progeny on the HP1 and HG8 bacterial lawn was significantly reduced compared to the BD24 non-toxic control (*p* < 0.0001). Results shown represent means ± standard error from triplicate samples.

There was no significant difference in the proportion of eggs hatching following exposure to HP1 or HG8 bacterial lawns compared to the non-toxic BD24 clone (one-way ANOVA followed by Tukey’s test; F (2, 6) = 0.4181; *p* = 0.6891 and *p* = 0.9908, respectively, Figure 2A). However, the L1 larvae fed on HP1 and HG8 bacteria did not survive past 24 h. To determine the impact of HP1 and HG8 on nematode brood size, a single *C. elegans* hermaphrodite was challenged against the toxic *E. coli* clones and the number of progenies resulting from the single nematode was quantified. A significant reduction in progeny was observed with both HP1 and HG8 strains compared to the number of progenies on the BD24 strain (one-way ANOVA followed by Tukey’s test; F (2, 6) = 169.7; *p* < 0.0001, Figure 2B). These findings suggest that both HP1 and HG8 did not affect *C. elegans* eggs’ hatching efficiency; however, they were toxic to young progeny which resulted in diminishing *C. elegans* brood size.

### 3.4. HP1::GFP and HG8::GFP Bacterial Strains Can Colonise and Persist in C. elegans Gastrointestinal System

**Figure 3:**
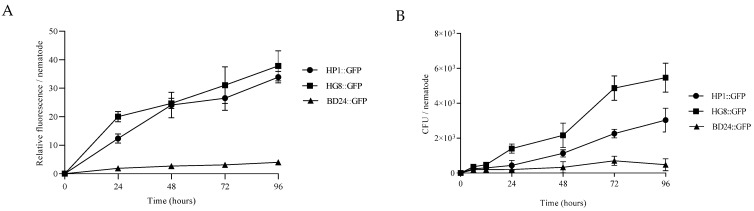
(**A**) Relative fluorescence representing the colonisation of HP1::GFP, HG8::GFP and the control strain BD24::GFP within *C. elegans* body. Data is presented as the mean of relative fluorescence from triplicate samples ± standard error. (**B**) Bacterial colonisation assay of HP1::GFP, HG8::GFP and BD24::GFP against *C. elegans*. Number of viable bacteria in the *C. elegans* gastrointestinal system is shown as colony-formation unit (CFU)/nematode. Data is presented as the mean of colony-formation unit per nematode (CFU/nematode) from triplicate samples ± standard error.

Quantification of GFP-labelled fluorescence after 24 h demonstrated a significantly higher degree of colonisation for strains HP1::GFP (*p* = 0.0044) and HG8::GFP (*p* = 0.0002) compared to the non-toxic BD24::GFP control strain (one-way ANOVA followed by Tukey’s test; F (2, 6) = 42.57, Figure 3A). The relative fluorescence increased for these strains over the course of the experiment but remained undetected for controls (Supplementary Materials, Figure S1) suggesting HP1 and HG8 can colonise and persist in the nematode gut. To more accurately quantify the number of HP1::GFP and HG8::GFP cells colonising the nematode gastrointestinal system, the nematode lysate was cultivated on LB10 agar. After 6 h of exposure to the bacterial strains, no significant differences were observed between the number of HP1::GFP and HG8::GFP bacterial colonies compared to BD24::GFP (one-way ANOVA followed by Tukey’s test; F (2, 6) = 2.586; *p* = 0.7434 and *p* = 0.1432, respectively, Figure 3B). However, the number of CFUs (colony-formation units) of HP1::GFP and HG8::GFP increased after 24 h. The highest number of HP1::GFP and HG8::GFP CFUs were counted after 96 h of exposure with a higher bacterial load in nematodes treated with HG8::GFP (one-way ANOVA followed by Tukey’s test; F (2, 6) = 44.09, *p* = 0.0090; Figure 3B). In nematodes exposed to HP1::GFP and HG8::GFP bacterial load increased 12-fold and 15-fold, respectively, at 96 h of exposure compared to the initial CFU at the 6-hour time point. In contrast, the number of the BD24::GFP strain increased only twofold after 96 h. This finding is in agreement with microscopic observation showing a higher abundance of HP1::GFP and HG8::GFP bacteria in the *C. elegans* gastrointestinal system compared to the negative control BD24::GFP strain (Supplementary Materials Figure S1).

### 3.5. Exposure to HP1::GFP and HG8::GFP Resulted in Morphological Changes in C. elegans

**Figure 4:**
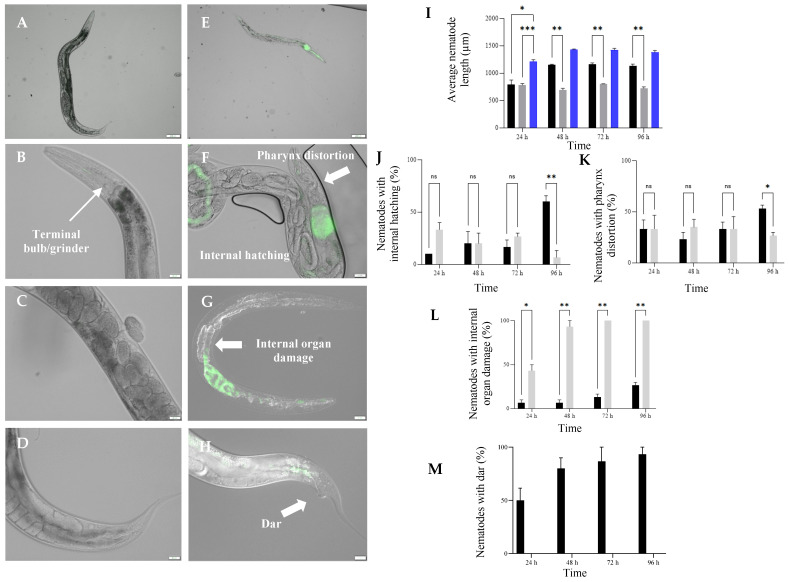
Examples of *C. elegans* body structure following exposure to HP1::GFP, HG8::GFP (**E**–**H**) and the non-toxic control BD24::GFP (**A**–**D**). Proportion of *C. elegans* with signs of morphological change after exposure to different GFP-tagged *E. coli* clones are shown in (**I**) (average nematode length), (**J**) (nematode with internal hatching), (**K**) (nematode with pharynx distortion), (**L**) (nematode with internal organ damage) and (**M**) (nematode with dar). Black, grey and blue bars indicate the proportion of morphological changes in *C. elegans* as a result of exposure to HP1::GFP, HG8::GFP and the non-toxic control BD24::GFP strains, respectively. Images of *C. elegans* were acquired using the DIC filter and the physical condition assessed for at least 30 worms per treatment. Graphical summaries represent the average percentage of assayed nematodes from triplicate samples ± standard error (*n* = 3). A *p* value < 0.05 was considered as statistically significant. Significance levels are indicated on figures as follows; * *p* < 0.05, ** *p* < 0.01, *** *p* < 0.001, ns *p* > 0.05.

Adult nematodes treated with the non-toxic control BD24::GFP showed a normal body length (~1.0 mm), a healthy pharynx, an intact internal organ, eggs without any evidence of internal hatching and a normal anal region (Figure 4A–D). Whilst a small accumulation of BD24::GFP cells was observed at the *C. elegans* pharynx lumen (indicated by faint green fluorescence before the grinder bulb), no morphological changes were observed within this region (Figure 4B). In contrast, *C. elegans* exposed to HP1::GFP and HG8::GFP strains showed evidence of physical changes including smaller body size, pharynx distortion, internal hatching and internal organ damage (Figure 4E–G). The growth of the *C. elegans* was retarded upon 24 h exposure to HP1::GFP and HG8::GFP indicated by body length reduction compared to the normal adult nematodes fed with BD24::GFP (two-way ANOVA followed by Tukey’s test; F (2, 6) = 179.4; *p* = 0.0441 and *p* = 0.0007, respectively, Figure 4A,E,I) and remained small after 96 h compared to the control nematodes. Furthermore, an increase in pharynx distortion was observed in *C. elegans* exposed to both HP1::GFP and HG8::GFP and the proportion was higher in nematodes treated with HP1::GFP following 96 h of exposure (two-way ANOVA followed by Tukey’s test; F (2, 6) = 17.36; *p* = 0.0088, Figure 4F,K). A higher proportion of internal hatching was also observed for *C. elegans* exposed to HP1::GFP compared to HG8::GFP after 96 h (two-way ANOVA followed by Tukey’s test; F (2, 6) = 17.36; *p* = 0.0088, Figure 4F,J). The proportion of nematodes with internal organ damage constantly increased until all of the nematodes exposed to HG8::GFP were damaged internally after 72 h (two-way ANOVA followed by Tukey’s test; F (2, 6) = 442.1; *p* = 0.0033, Figure 4G,L). Unlike the other morphological changes, a deformed anal region (dar) was only observed on *C. elegans* exposed to HP1::GFP (Figure 4H). Half of the HP1::GFP exposed- *C. elegans* displayed a dar morphology after 24 h and the proportion of nematodes showing dar increased until at 96 h ~93% of the nematodes had the dar morphology (two-way ANOVA followed by Tukey’s test; F (2, 6) = 58.84; *p* = 0.0092 when compared to HG8::GFP, Figure 4M).

### 3.6. Exposure to HP1::GFP and HG8::GFP Results in Loss of C. elegans Cell Membrane Integrity

**Figure 5:**
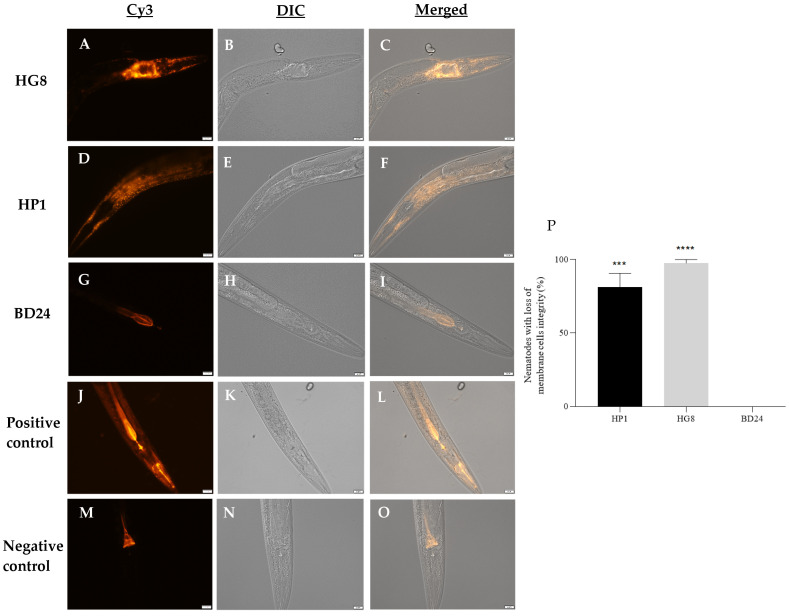
Propidium iodide (PI) staining on *C. elegans* exposed to HG8 (**A**–**C**), HP1 (**D**–**F**) and BD24 (**G**–**I**) strains. PI staining on nematodes exposed to excessive heating (**J**–**L**) and nematodes fed on *E. coli* OP50 (**M**–**O**) were used as the positive and negative controls, respectively. Nematode images were acquired under 40× magnification using the Cy3 filter (**A**,**D**,**G**,**J**,**M**) and DIC filters (**B**,**E**,**H**,**K**,**N**). The captured images were then merged to give a precise visualisation of PI staining within the body of *C. elegans* (**C**,**F**,**I**,**L**,**O**). Scale bars indicate 20 µm. The proportion of *C. elegans* showing loss of cell membrane integrity after 48 h of exposure to HP1 and HG8 bacteria was indicated in (**P**). No loss of cell membrane integrity was observed on *C. elegans* treated with the negative control BD24. Results are representative data of mean percentage of nematodes showing loss of cell membrane cells integrity from triplicate assays ± standard error. *** denotes *p* = 0.0001, **** denotes *p* < 0.0001.

Pharyngeal and intestinal cells of nematodes exposed to the HP1 and HG8 strains showed evidence of a loss of cell membrane integrity indicated by the uptake of propidium iodide (Figure 5A–F). In total, ~81% and ~97% of nematodes treated with HP1 and HG8 strains, respectively, showed loss of cell membrane integrity, whereas none of the nematodes exposed to control strain BD24 showed evidence of the phenotype (Figure 5P).

### 3.7. HP1 and HG8 Protein Extracts Are Toxic against C. elegans

**Figure 6:**
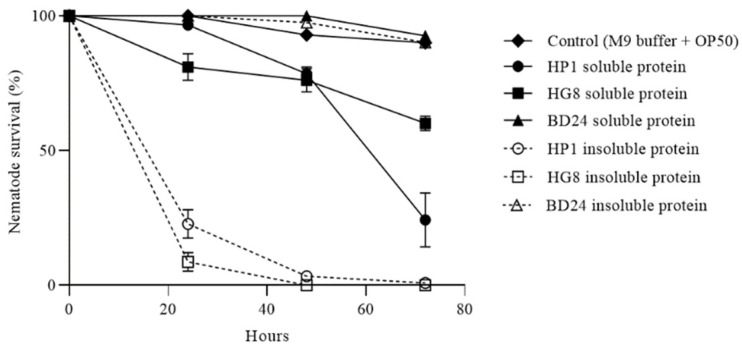
*C. elegans* survival in the soluble and insoluble protein fractions of HP1, HG8 and BD24 using the 24-well microtiter plates. The total protein in each fraction was ~0.7 mg/mL. The non-toxic BD24 protein fractions and M9 buffer added with *E. coli* OP50 were used as the negative controls. Exposure to the HP1 and HG8 protein extracts reduced nematode survival with the decrease more pronounced in the insoluble compared to the soluble protein fraction for each strain (*p* < 0.0001). Each data point represents means of nematode survival ± standard error from triplicate samples.

Despite HP1 and HG8 bacterial cell-free supernatant being non-toxic to *C. elegans* (data not shown), soluble and insoluble protein fractions were assessed for nematode-killing activity. After 48 h, treatment with HP1 and HG8 protein fractions resulted in diminished *C. elegans* survival with toxic activity being more pronounced in the insoluble protein fraction (one-way ANOVA followed by Tukey’s test; F (6, 14) = 427.2; *p* < 0.0001, Figure 6). In contrast, *C. elegans* treated with the soluble and insoluble protein fractions of the non-toxic BD24 control clone had no impact on nematode survival (one-way ANOVA followed by Tukey’s test; F (6, 14) = 427.2; *p* = 0.9783, Figure 6). These results suggest that the nematode-killing activity likely results from a proteinaceous compound associated with HP1 and HG8 bacterial cells. The toxic compound is hereafter denoted ‘nematode-killing protein-1’ (Nkp-1).

### 3.8. Nkp-1 Treatment Causes Physical Damage to C. elegans Cells

**Figure 7:**
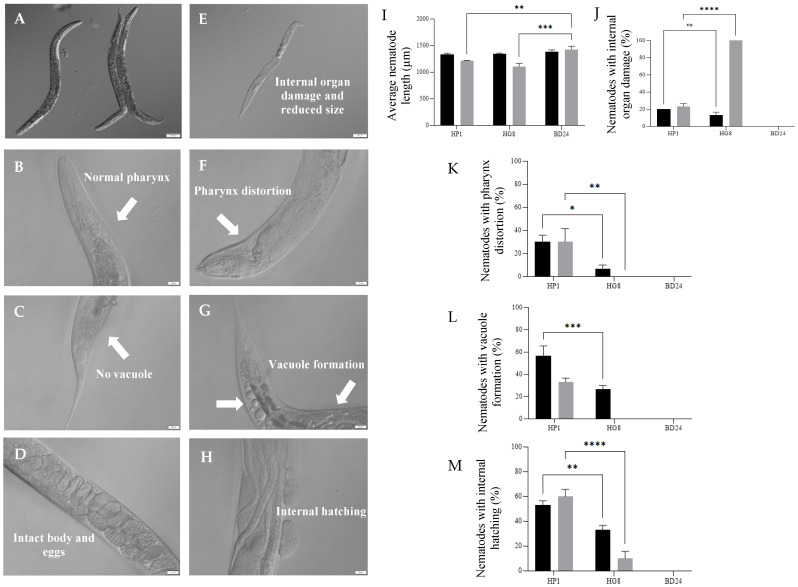
Examples of physical appearances observed on *C. elegans* treated with the total insoluble protein fractions of non-toxic BD24 (**A**–**D**) and Nkp-1 expressing strains (HP1 and HG8). Several morphological changes, i.e., reduced body size based on the average nematode length (**E**,**I**), internal organ damage (**E**,**J**), pharynx distortion (**F**,**K**) vacuole formation (**G**,**L**) and internal hatching (**H**,**M**) were observed for nematodes exposed to protein fractions of HP1 and HG8. Results shown are representative data from the mean percentage of nematodes with morphological changes from triplicate samples ± standard error. A *p* value < 0.05 was considered statistically significant. Soluble protein: black; insoluble protein: grey. Significance levels are indicated on figures as follows: * *p* < 0.05, ** *p* < 0.01, *** *p* < 0.001, **** *p* < 0.0001, ns *p* > 0.05.

While *C. elegans* exposed to BD24 protein extract displayed a normal morphology (Figure 7A–D), nematodes exposed to either the soluble or insoluble protein of HP1 and HG8 strains after 72 h showed several morphological changes, i.e., body length reduction (indicating growth retardation), internal organ damage, pharynx distortion, vacuole formation and internal hatching (Figure 7E–H). The insoluble proteins from both clones negatively impact nematode growth (two-way ANOVA followed by Tukey’s test; F (1, 12) = 10.47; *p* = 0.0059 and *p* = 0.0002, respectively, Figure 7E,I) while treatment with the insoluble protein of HP1 resulted in a higher proportion of nematodes with pharynx distortion compared to nematodes exposed to protein extracts from HG8 (two-way ANOVA followed by Tukey’s test; F (1, 12) = 0.2500; *p* = 0.0262 and *p* = 0.0056, respectively, Figure 7K). An increase in vacuole formation was also observed for nematodes treated with the soluble protein of HP1 compared to the soluble protein of HG8 (two-way ANOVA followed by Tukey’s test; F (1, 12) = 25.00; *p* = 0.0006, Figure 7L). In addition, exposure to both protein fractions from HP1 resulted in a higher proportion of internal hatching in *C. elegans* compared to exposure to either protein fraction from HG8 (two-way ANOVA followed by Tukey’s test; F (1, 12) = 3.125, *p* = 0.0083 and *p* < 0.0001, Figure 7M).

### 3.9. Nkp-1 Harbours a Carbohydrate-Binding Module 

Protein bands corresponding to the estimated size of Nkp-1 (~25 kDa) were observed through SDS PAGE in the protein fractions of HP1 and HG8 strains (Supplementary Materials, Figure S2). These protein bands were excised from the gel and identified as Nkp-1 using LC-MS analysis. 

**Figure 8:**
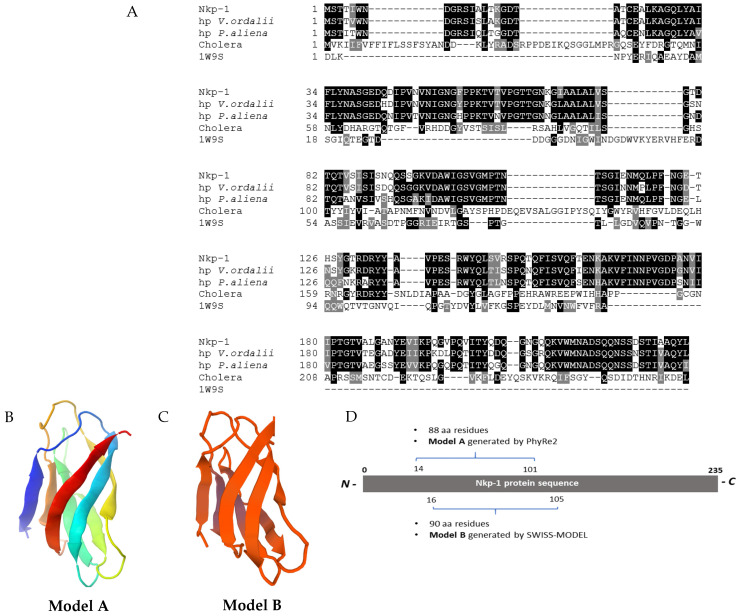
(**A**) Multiple sequence alignment of Nkp-1 protein sequence and closely related protein sequences, including the uncharacterised hypothetical protein (hp) from *V. ordalii* and *P. aliena* and characterised protein sequences; Cholera Enterotoxin Subunit A from *V. cholerae* (denoted as Cholera) and a hypothetical protein BH0236 from *B. halodurans* (PDB ID: 1W9S) harbouring a β-1,3-glucan binding CBM6 module [54] (denoted as 1W9S). The total amino acid residues for each of the protein sequences are: Nkp-1 (235 aa), hp *V. ordalii* (235 aa), hp *P. aliena* (235 aa), Cholera (258 aa) and 1W9S (1020 aa). The consensus residues in each aligned protein sequence are highlighted in black and grey whilst the identified gaps may indicate deletion or insertion of amino acids. The sequence alignment was created using T-Coffee [56] and displayed using Boxshade (ExPASy). (**B**) Model A Nkp-1 was generated by PhyRe2 using the structure of beta-1,3-glucan binding CBM6 module of hypothetical protein BH0236 from *Bacillus halodurans* (PDB ID: 1W9S). The model dimensions (Å): X:26.001 Y:40.693 Z:33.630 indicates the permitted rotation of the C-N bond in the polypeptide chain. (**C**) Model B Nkp-1 was generated by SWISS-MODEL using the structure of PsCBM35-2 domain containing carbohydrate-binding module-35 (CBM35) of α-1,6-glucosyltransferase from *Paenibacillus* sp. 598K (PDB ID: 5X7O). The protein alpha helices are shown as arrows whilst the beta strands are shown as ribbons. The coil and turn protein are indicated in line. The arrowhead points towards the carboxy termini. (**D**) Nkp-1 protein coverage that is used by PhyRe2 and SWISS-MODEL to generate the Nkp-1 3D protein model A and B, respectively.

The Nkp-1 protein sequence (consisting of 235 aa) was aligned to the sequences of closely related homologs of Nkp-1 (obtained from the BLASTp results, see Figure 8A) including the cholerae toxin from *Vibrio cholerae* [52,53] and a hypothetical protein from *Bacillus halodurans* (PDB ID: 1W9S) harbouring the carbohydrate-binding module [54]. Homology modelling of Nkp-1 resulted in a 3D protein model (Figure 8B) based on the highest confidence scoring template, a β-1,3-glucan binding CBM6 module (galactose-binding domain-like, family 6 carbohydrate-binding module) of hypothetical protein BH0236 from *B. halodurans* (PDB ID: 1W9S) [54]. In total, 88 residues of Nkp-1 from residue 14 to residue 101 (37% of the Nkp-1 sequence) located in the N-terminal of the protein were modelled with 48% confidence (Figure 8D). Over half (56%) of the Nkp-1 aligned sequence were modelled as having a secondary structure consisting of anti-parallel beta strands or β barrel, which is composed of tandem repeats that coil and twist to construct a closed toroidal structure. A second 3D model was built using SWISS-MODEL based on the PsCBM35-2 domain containing carbohydrate-binding module 35 (CBM35) of α-1,6-glucosyltransferase (classified under the glycoside hydrolase (GH) family 31 alpha-glucosidase) from *Paenibacillus* sp. 598K (PDB ID: 5X7O) [55] (Figure 8C). The alignment resulted in 23.8% identity from residue 16 to residue 105 (Figure 8D).

## 4. Discussion

### 4.1. HP1 Is Responsible for Nkp-1 Expression 

Sequencing and gene complementation of non-toxic mutant 7C8 revealed that the expression of a single gene, *HP1*, was sufficient for toxic activity against *C. elegans*. The *HP1* encodes for a novel nematode-killing protein (Nkp-1), which is toxic to *C. elegans* in both soluble and insoluble forms. While the intact cells of Nkp-1-expressing *E. coli* were active against *C. elegans*, corresponding cell-free supernatants were non-toxic, suggesting that the concentration of Nkp-1 in the supernatant is too dilute or that the protein is not secreted to the environment. Other studies have observed nematode-killing compounds that are retained within the cell of the producers. For example, prodigiosin in *Serratia marcescens* cell extracts is toxic against the plant parasitic nematodes; *Radopholus similis* and *Meloidogyne javanica* [57] and the insoluble nematicidal Cry5B crystal protein was also purified from the intact cells of *Bacillus thuringiensis* [58]. 

Analysis of the Nkp-1 protein sequence led to the generation of two models (Model A and B) both consisting of one of two different putative carbohydrate-binding modules (CBMs) (i.e., CBM6 or CBM35) (Figure 8B,C). The structural features of both carbohydrate-binding modules are composed of canonical β-sandwich folds which are connected by loops of different length (http://www.cazy.org/. Accessed on 20 May 2020) [59]. The CBM6 and CBM35 carbohydrate-binding modules are found in several enzymes, i.e., endoglucanase, β-agarase, α-1,3-glucanase, glucuronoxylanase, esterase and mannanase [60,61,62,63,64] and bacterial toxins, i.e., crystal toxins (Cry) from *Bacillus thuringiensis* [65,66]. These carbohydrate-binding modules enable enzymes to bind to the target substrate [67] or toxins to bind to the membrane or glycoconjugate receptors of the target host cells [65,68]. As an example, insoluble Cry proteins produced by *B. thuringiensis* are potent nematicidal and pesticidal toxins that harbour the CBM6 and CBM35 carbohydrate-binding module [66,69]. The Cry toxins generally consist of three structural domains, where domain I is responsible for protein toxicity, domain II is associated with toxin binding to the host glycoconjugate receptor and domain III is important for both host receptor recognition and toxin binding [65,70]. 

The Nkp-1 models harbouring the CBM6 (Figure 8B) and CBM35 modules (Figure 8C) consist of an antiparallel β-sandwich protein arrangement. This β-sandwich structure is thought to be key to the specificity of Cry toxin binding to the target nematode and insect’s intestinal cell receptor [65,71], with studies showing that swapping this structure between different Cry toxins results in altered specificity and toxicity of the toxin towards several target organisms [72,73]. The β-sandwich structure (domain III) is also important for Cry protein structural integrity and stability due to the network of hydrogen bond and van der Waals interactions between the protein residues and the side chains [71,73] Given the similar arrangement of antiparallel β-sandwich in Nkp-1, it is possible that these carbohydrate-binding modules play a role in recognition and binding to one of the many glycoconjugate receptors present in the *C. elegans* intestine [68,74]. While the specific Nkp-1-targetting glycoconjugate receptor in *C. elegans* warrants further investigation, this discovery suggests the initial mechanism of Nkp-1 killing activity against *C. elegans* involves attachment to the host membrane receptor, prior to the initiation of nematicidal activity.

### 4.2. Nkp-1 Expressing E. coli Clones Kill C. elegans via a Proposed Step-by-Step Mode of Action (MOA)

**Figure 9:**
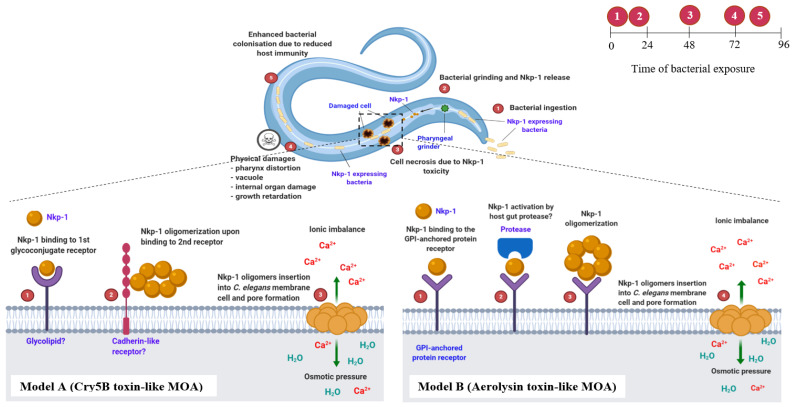
Schematic diagram representing the proposed mode of action (MOA) of Nkp-1 expressing bacteria (HP1 and HG8) against *C. elegans*. Above, the proposed step by step (numbers 1 to 5; circled in red) nematode-killing mechanism of Nkp-1 expressing bacteria in *C. elegans* body and the time frame (**top right**) of each Nkp-1 intoxication phase (starting from 0 h to 96 h of bacterial exposure). Below, the proposed models A and B of Nkp-1 MOA in causing necrotic cell death in *C. elegans* gastrointestinal system; see text for more details. Image created with BioRender.com.

We observed that ingestion of Nkp-1 expressing bacterial strains results in morphological damage and cellular injury (necrosis) to the nematodes, leading to the animal’s death. A step-by step mode of action model has been proposed to show the killing activity of Nkp-1 expressing bacteria against *C. elegans* (see Figure 9). 

**Stage 1:** Ingestion and digestion of Nkp-1 expressing bacteria by *C. elegans*. When the pharyngeal muscles contract, bacteria are ingested, concentrated, pulverized (using a “grinder” located in the terminal bulb) and passed into the intestine through the pharyngeal-intestinal valve for degradation and nutrient absorption [75,76]. However, upon ingestion of toxic bacteria, including HP1 and HG8, this grinding activity may also result in the liberation of toxic metabolites, such as Nkp-1 (Figure 9). While future work is required to demonstrate the release of Nkp-1 by the nematode pharyngeal grinder, similar mechanisms have previously been demonstrated for the release of Cry toxin from *B. thuringiensis* [42,77]. In addition, nematode species lacking the pharyngeal grinder bulb, i.e., *Pristionchus pacificus*, are resistant to *B. thuringiensis* producing the Cry toxin [78] further suggesting that pharyngeal grinding in *C. elegans* may enable the release of Nkp-1 from both HP1 and HG8 strains. 

**Stage 2:** Nkp-1 triggers cellular level damage (necrosis) in *C. elegans*. Upon bacterial ingestion and Nkp-1 liberation, one possibility is that the toxic protein functions as a degradative enzyme targeting *C. elegans* body components; however, as there was no evidence of lipase, protease, chitinase or gelatinase/collagenase activity, an Nkp-1 killing mechanism via degradative enzymatic activity is unlikely. Rather, microscopic observation of nematodes exposed to the Nkp-1 expressing clones suggested that Nkp-1 may function as a pore-forming toxin (PFT) causing necrotic cell death (necrosis), as intestinal cells were permeable to propidium iodide (PI) staining (Figure 5A–F,P) [79,80]. 

Two different models, Cry5B-like (based on Cry toxin by *B. thuringiensis*) and aerolysin toxin-like (based on aerolysin toxin by *Aeromonas* sp.) (Figure 9) [77,81], are proposed to describe the Nkp-1 mode of action. In both models, the Nkp-1 protein is solubilised in the nematode intestine prior to it binding to a yet undetermined glycoconjugate receptor of intestinal cells, for example, the invertebrate-specific glycolipid in Model A [77] or the glycosyl phosphatidyl inositol (GPI)-anchored protein in Model B [81,82] (Figure 9). To activate toxicity, aerolysin needs to be cleaved at the C-termini by the host gut protease [81,83]. However, whether Nkp-1 requires proteolytic activation is still unknown. Next, as occurs for both Cry5B and aerolysin toxins, the Nkp-1 is predicted to oligomerize, generating a ring-like structure inserted into the nematode intestinal cells hence resulting in pore formation (Figure 9). For β-PFT toxins harbouring an anti-parallel β-barrel (as displayed by the Nkp-1 3D protein model; see Figure 8B,C) toxin oligomerisation is a crucial step prior to the oligomers’ insertion into the targeted host cell [83]. There is evidence that the cadherin-like receptor in *C. elegans* facilitates the oligomerization of Cry5B toxin and its formation of pores [84]. However, whether the cadherin-like receptor is required for Nkp-1 oligomerization and pore formation warrants further investigation. Following the insertion of Nkp-1 oligomers, it is proposed that the plasma membrane of nematode intestinal cells is punctured, resulting in vacuole formation due to osmotic imbalance between the cytoplasm and the extracellular environment followed by necrosis and physical damage (Figure 9) [42,77,84].

**Stage 3:** Nkp-1-expressing bacteria cause physical damage to *C. elegans*. Exposure of Nkp-1 to *C. elegans* results in severe growth retardation (see Figure 7E,I). Consistent with this finding, toxic proteins Pp-ANP1a and Pp-ANP2a produced by *Pseudomonas protegens* and aflatoxin by *Aspergillus flavus* and *Aspergillus parasiticus* also caused growth retardation in *C. elegans* [27,85]. Similarly, exposure to *E. coli* clones expressing the *B. thuringiensis* Cry21Fa1 or Cry21Ha1 toxins results in reduced body size in *C. elegans* [86]. Nematode growth retardation upon exposure to these toxins could be a result of reduced nutrient absorption efficiency caused by the perturbed intestinal cells and damaged micro villi [87]. An increase in pharynx distortion was also observed on *C. elegans* exposed to the Nkp-1-expressing clones and their protein extracts (Figure 4F,K and Figure 7F,K). Pharynx distortion has been associated with “rigor mortis” and increasing cytoplasmic calcium (Ca^2+^) which are the early signs of dying muscle cells due to necrosis in the *C. elegans* pharyngeal and gastrointestinal system [88]. Whilst pharynx distortion could be an indicator of ageing in nematodes, it also can occur as an immediate impact of bacterial evasion of pharyngeal cells (particularly at the terminal bulb), resulting in nematode mortality [89]. Indeed, other studies have shown an increase in this phenotype because of uncontrolled bacterial proliferation [90]. Vacuole formation and internal body damage (indicated by excessive physical discolouration, shrinking intestine and degenerated gonadal system and germline cells) was also observed in *C. elegans* exposed to the Nkp-1-expressing clones and their protein extracts (Figure 4G and Figure 7E,G). Vacuole formation, which may represent swelling necrotic-like cell death [91], is strongly linked to necrosis in *C. elegans* [92]. Similar phenotypes have been observed for nematodes exposed to microbial pathogens [93,94] and other toxins, including pore-forming toxins (PFTs) such as Cry toxin produced by *B. thuringiensis* [95] and cytolysin produced by *V. cholerae* [93,96]. 

**Stage 4:** Enhanced Nkp-1-expressing bacterial colonisation. The intestine not only acts as a site for nutrient absorption but also provides important protective immunity against harmful microorganisms [97,98]. Given the important immune function of the intestine, severe intestinal damage as a result of exposure to Nkp-1 may increase the susceptibility of *C. elegans* to bacterial proliferation and deteriorate its fitness. Indeed, both direct microscopic observation and bacterial CFU counts indicated that *E. coli* cells expressing Nkp-1 are able to proliferate in the intestine of *C. elegans* (Figure 3, Supplementary Materials Figure S1). This observation could be linked to the MOA of bacteria producing protein toxins, i.e., indirectly hijacking the host immune system and its antimicrobial effectors hence enhancing bacterial colonisation of the host [99]. The result of this proposed MOA for Nkp-1 described above is *C. elegans* mortality.

### 4.3. Dar Formation and Internal Hatching; C. elegans Response against Nkp-1 Expressing Bacteria

Internal egg hatching was also observed in *C. elegans* individuals exposed to the Nkp-1 expressing strains or their corresponding protein extracts (Figure 4F,J and Figure 7H,M). This phenotype has been observed in *C. elegans* challenged with *Vibrio parahaemolyticus* and when sequentially exposed to *Staphylococcus aureus* and *Proteus mirabilis* [100,101] Internal hatching allows the eggs to hatch within parental nematodes and acts to protect progeny against starvation and/or bacterial toxicity [102,103]. However, this phenotype adversely diminished the survival of the parent nematode by damaging the gonads resulting in matricidal death [102]. Therefore, it is possible that internal egg hatching occurs as part of a general stress response to Nkp-1 and could be used by *C. elegans* as a mechanism of maintaining its population.

Notably, the deformed anal region (dar) phenotype or swelling of the anal region was only observed in *C. elegans* exposed to the HP1 strain (Figure 4H,M). Previous studies have suggested that the dar phenotype acts as an immune response against bacteria as mutants lacking this phenotype experienced high rates of infection [104,105] Dar formation has also been observed in *C. elegans* exposed to several other microbes including *Microbacterium nematophilum* [104], *Saccharomyces cerevisiae* [105] and *Coxiella burnetii* [106]. Unlike *M. nematophilum*, which was abundantly found adhering the nematode rectum [107], HP1 colonises the *C. elegans* intestinal lumen particularly at the anterior region. Similarly, *C. elegans* exposed to *S. cerevisiae* and *C. burnetii* also demonstrated intestinal lumen colonisation but without cell accumulation at the nematode rectum [105,106] It has been previously suggested that the dar phenotype is a mechanism employed by *C. elegans* to ease bacterial removal from the anal opening through the epithelial rectal swelling mechanism [104,105,108]. In addition, nematodes with dar also show an avoidance behaviour against the toxic bacteria that may indirectly facilitate the clearance of toxic bacterial infections from the intestine [108]. Interestingly, the dar phenotype was not seen in *C. elegans* exposed to HG8. One possible explanation for this observation is that the HP1 bacterial colonisation and its toxic effect is milder compared to the HG8 strains. This observation could be linked to the higher abundance of HG8 bacterial cells in *C. elegans* compared to the HP1 strain within 24 h of bacterial exposure (see Figure 3A,B, Supplementary Materials, Figure S1). Consequently, within 24 h, while 50% of HP1-exposed nematodes showed the dar phenotype, almost half of the HG8-exposed nematodes were severely damaged leading to their rapid death.

## 5. Conclusions

This study has identified the previously uncharacterised protein Nkp-1 as the causative agent for the nematode fast killing activity of the marine bacterium *P. tunicata*. Nkp-1 does not appear to have any enzymatic activity; rather, based on the modelling of a carbohydrate-binding domain, Nkp-1 is hypothesised to act as a novel pore-forming toxin (PFT). Histological observations of *C. elegans* individuals exposed to Nkp-1, including loss of membrane integrity and necrosis, support the theory of a PFT mode of action. Severe physical damages observed on *C. elegans* particularly in the intestinal region may reduce nematode immunity, resulting in increased colonisation by the Nkp-1 expressing bacteria and a shortened nematode lifespan.

Whilst the use of Nkp-1 as a future nematicide is promising, more work is required to fully understand its mode of action and safety. For example, the comparative analysis of Nkp-1 to the current nematicide; BT toxin (i.e., Cry5B), via dose-response assay and the analysis of Nkp-1’s efficacy against the Cry BT toxin-mutant, i.e., *bre* nematodes, to confirm its potential binding to the nematode glycoconjugate receptor. Importantly, while *C. elegans* represents a good model system, future investigation of Nkp-1 toxicity against human (e.g., *Ancylostoma duodenale*, *Brugia malayi*), animal (e.g., *Anisakis simplex*, *Haemonchus contortus*) or plant parasitic nematodes (e.g., *Meloidogyne incognita*, *Radopholus similis*) should be performed in vitro and in vivo to understand the broad-scale potential of this new anti-nematode agent. Finally, the cytotoxicity of Nkp-1 in host cell lines and non-target organisms will be an important next step to determine Nkp-1’s suitability as an anthelminthic drug. Given the rapid increase in nematode drug resistance, it is hoped that the work presented here will facilitate future developments of Nkp-1 as a novel anti-nematode drug or biopesticide.

## Figures and Tables

**Table 1:** Bacterial strains and vectors used in this study.

Strain or Vector	Relevant Characteristic or Genotype	Reference or Source
Strain		
*E. coli*		
EPI300-T1R	F-*mcr*A ∆(*mrrhsd*RMS*mcr*BC) Φ80d*lac*Z∆M15∆*lac*X74 *rec*A1 *end*A1 *ara*D139 ∆(*ara*, *leu*) 7697 *gal*U *gal*K λ-*rps*L *nup*G *trf*A *ton*A *dhfr*	Epicentre
EPI300	F-*λ*-*mcr*A Δ(*mrr*-*hsd*RMS*-mcr*BC*)* Φ80*dlac*ZΔM15 Δ*(lac)*X74 *recA1 end*A1 *ara*D139 Δ*(ara, leu)7*697 *gal*U *gal*K *rps*L *(Str*R*) nup*G’ *trf*A *dhfr*	Epicentre
OP50	Uracil auxotroph	[20]
DH5α	ϕ80*dlac*ZΔM15 Δ(*lac*ZYA*-arg*F)U169 *rec*A1 *end*A1 *hsd*R17 (rk– mk+) *sup*E44 *thi*-1 *gyr*A *rel*A1, carrying pBAD24 vector	CMSI, UNSW
HP1	EPI300 transformed with pBAD24*HP1* (NCBI Accession: ZP_01132246.1)	This study
BD24	EPI300 transformed with empty pBAD24 vector	This study
HG8	EPI300-T1R transformed with pCC1FOS™::ZP_ 01132230.1 to ZP_ 01132246.1	[18]
HP1::GFP	HP1 transformed with p519ngfp plasmid	This study
BD24::GFP	BD24 transformed with p519ngfp plasmid	This study
HG8::GFP	HG8 transformed with p519ngfp plasmid	This study
7C8	HG8 transposon mutant library showing mutation at *HP1*	[21]
7C8::*HP1*	HG8 transposon mutant library showing mutation at ZP_01132246.1, complemented with pBAD24*HP1*	This study
7C8::pBAD24	HG8 transposon mutant library showing mutation at ZP_01132246.1, complemented with empty pBAD24 vector	This study
*P. aeruginosa* ATCC 9027	Clinical sample	American Type Culture Collection (ATCC^®^)
Vector		
pCC1FOS™::ZP_ 01132230.1 to ZP_ 01132246.1 ^a^	Fosmid backbone for genomic library of *Pseudoalteromonas tunicata* D2 carrying a wild type D2 insert (13.8 kb) expressing putative anti-nematode activity, Cm^r^	[18,22]
pCC1FOS™::ZP_ 01132230.1 to ZP_01132246.1 with EZ-Tn5™ Δ ZP_01132246.1 ^a^	HG8 fosmid mutated by EZ-Tn5™ transposon on *P. tunicata* wild type gene ZP_01132246, Cm^r^, Kan^r^	[21]
pBAD24 ^a^	F-, Δ(*arg*F-*lac*)169, φ80*dlac*Z58(M15), *gln*X44(AS), *λ*^−^, *rfb*C1, *gyr*A96(NalR), *rec*A1, *end*A1, *spo*T1, *thi*E1, *hsd*R17, pBAD24	[23]
pBAD24*HP1* ^a^	*P. tunicata* D2 wild type gene (ZP_01132246.1) cloned downstream the pBAD promoter, Amp^r^	This study
p519ngfp	High-copy-number plasmid with constitutive GFP expression; Km^r^	[24]

^a^ Inducible expression with the presence of L-arabinose 0.2% (*w*/*v*). CMSI denotes Centre for Marine Science and Innovation, University of New South Wales (UNSW).

## Data Availability

The data presented in this study are available within this article and the Supplementary Materials.

## Supplementary Materials

The following are available online at https://www.mdpi.com/article/10.3390/biomedicines9111586/s1, Figure S1: HP1::GFP and HG8::GFP bacterial colonisation within C. elegans gastrointestinal system, Figure S2: SDS polyacrylamide gel (12%) showing soluble and insoluble protein fractions of HP1, HG8 and the control strains; BD24 and EPI300.
