# Supplementary material for: Novel Nematode-Killing Protein-1 (Nkp-1) from a Marine Epiphytic Bacterium Pseudoalteromonas tunicata
Source: Biomedicines. 2021 Oct 30;9(11):1586. doi: 10.3390/biomedicines9111586 (PMC8615270; doi:10.3390/biomedicines9111586)
Supplement: Supplementary file 1 [file biomedicines-09-01586-s001.zip › biomedicines-1424168-supplementary.pdf]

Novel Nematode-killing Protein-1 (Nkp-1) from a Marine Epiphytic Bacterium *Pseudoalteromonas tunicata*

Nor Hawani Salikin [1,2,*], Malak Dubois [3], Jadranka Nappi [1], Helene Lebhar [3], Christopher Marquis [3] and Suhelen Egan [1,*]

[1] Centre for Marine Science and Innovation, School of Biological, Earth and Environmental Sciences, UNSW, Sydney, NSW 2052, Australia; j.nappi@unsw.edu.au
[2] School of Industrial Technology, Universiti Sains Malaysia, USM, Gelugor 11800, Penang, Malaysia
[3] School of Biotechnology and Biomolecular Sciences, UNSW, Sydney, NSW 2052, Australia;
malak.dubois@det.nsw.edu.au (M.D.); h.lebhar@unsw.edu.au (H.L.); c.marquis@unsw.edu.au (C.M.)
* Correspondence: norhawani@usm.my (N.H.S.); s.egan@unsw.edu.au (S.E.)

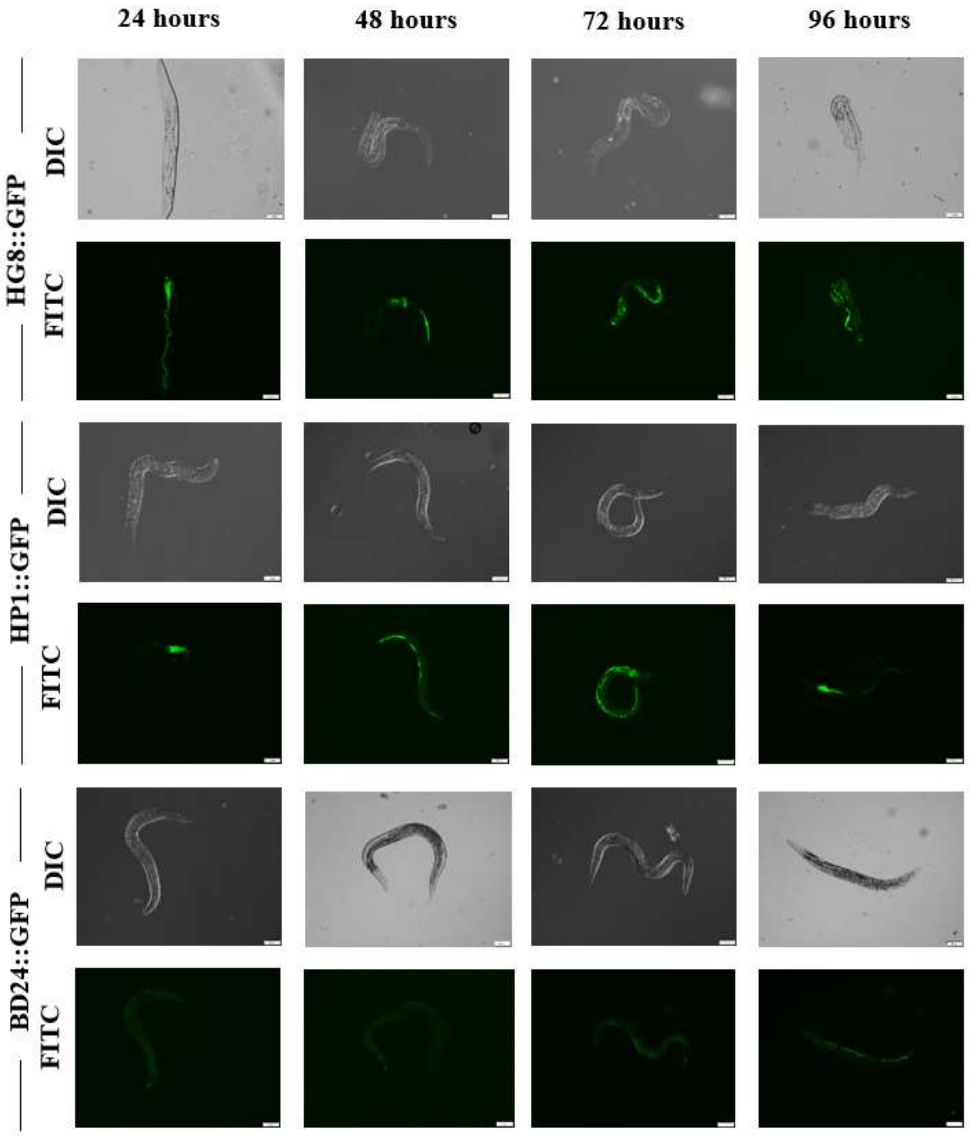

Figure S1. HP1::GFP and HG8::GFP bacterial colonisation within *C. elegans* gastrointestinal system. The BD24::GFP strain was used as the negative control. Nematode images were acquired under the 10x magnification (for HP1::GFP and BD24::GFP bacterial colonisation) and 20x magnification (for HG8::GFP bacterial colonisation) using the Olympus BLX-61 equipped with the DIC and FITC filter. Scale bars indicates 100 μm (for images of HP1::GFP and BD24::GFP-treated nematodes) and 50 μm (for images of HG8::GFP-treated nematodes). Bacterial colonisation is indicated by green fluorescence emitted by the GFP-tagged bacterial cells.

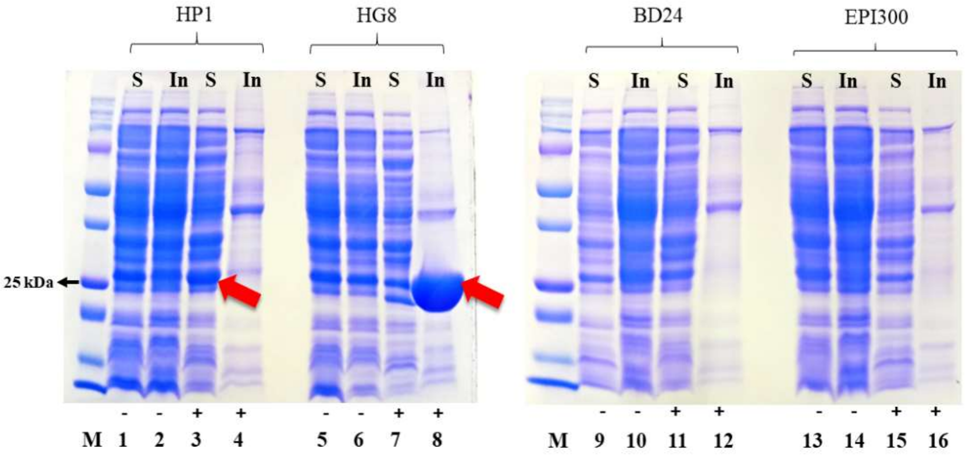

Figure S2. SDS Polyacrylamide gel (12%) showing soluble and insoluble protein fractions of HP1, HG8 and the control strains; BD24 and EPI300. Key: S; soluble protein fraction, In; Insoluble protein fraction, -; no L-(+)-Arabinose induction, + ; induced with L-(+)-Arabinose (0.2% w/v). Protein bands ~ 25.17 kDa (indicated by red arrows) were confirmed as the Nematode killing protein-1 (Nkp-1) encoded by *P. tunicata* D2 *hp1* (NCBI Accession: ZP_01132246.1). The Nkp-1 was not detected in the protein fractions of BD24 or EPI300 bacterial strains.
